# Delayed Monocular SLAM Approach Applied to Unmanned Aerial Vehicles

**DOI:** 10.1371/journal.pone.0167197

**Published:** 2016-12-29

**Authors:** Rodrigo Munguia, Sarquis Urzua, Antoni Grau

**Affiliations:** 1 Department of Computer Science, CUCEI, University of Guadalajara, Guadalajara, México; 2 Automatic Control Dept, Technical University of Catalonia, 08034 Barcelona, Spain; Beihang University, CHINA

## Abstract

In recent years, many researchers have addressed the issue of making Unmanned Aerial Vehicles (UAVs) more and more autonomous. In this context, the state estimation of the vehicle position is a fundamental necessity for any application involving autonomy. However, the problem of position estimation could not be solved in some scenarios, even when a GPS signal is available, for instance, an application requiring performing precision manoeuvres in a complex environment. Therefore, some additional sensory information should be integrated into the system in order to improve accuracy and robustness. In this work, a novel vision-based simultaneous localization and mapping (SLAM) method with application to unmanned aerial vehicles is proposed. One of the contributions of this work is to design and develop a novel technique for estimating features depth which is based on a stochastic technique of triangulation. In the proposed method the camera is mounted over a servo-controlled gimbal that counteracts the changes in attitude of the quadcopter. Due to the above assumption, the overall problem is simplified and it is focused on the position estimation of the aerial vehicle. Also, the tracking process of visual features is made easier due to the stabilized video. Another contribution of this work is to demonstrate that the integration of very noisy GPS measurements into the system for an initial short period of time is enough to initialize the metric scale. The performance of this proposed method is validated by means of experiments with real data carried out in unstructured outdoor environments. A comparative study shows that, when compared with related methods, the proposed approach performs better in terms of accuracy and computational time.

## 1 Introduction

There are still important problems to be solved in autonomous robotics, and simultaneous localization and mapping (SLAM) is one of them. This paper tries to tackle this problem and contributes to give even more autonomy to mobile robots. Regarding the term SLAM, it is used to refer to a map building process in an unknown space and the use of this map to navigate through such an space tracking the position in a simultaneous process. Usually this map is built using the sensors that the device (an aerial vehicle in this case) have on board, (see [1, 2] for a complete survey).

Many different kinds of sensors can be used for implementing SLAM systems, for instance, laser ([3–5]), sonar ([6–8]), sound sensors ([9, 10]), RFID ([11, 12]) or computer vision ([13–15]). The selection of such a sensor technology has a great impact on the algorithm used in SLAM and, depending on the application and other factors, each technology has some strong and weak points.

This work proposes a novel vision-based SLAM method to be applied to a quadcopter. In the case of small unmanned aerial vehicles (UAVs), there exist several limitations regarding to the design of the platform, mobility and payload capacity that impose considerable restrictions on the available computational and sensing resources. Recently, the availability of lighter laser range finders has allowed the use of this kind of sensors in small UAVs. Some examples of SLAM systems with application to UAVs that make use of laser range finders are: [16, 17] and [18]. While a good performance can be obtained with laser range finders, video cameras still represent an excellent choice for its use in small UAVs. Those devices provide many data and can be hardware-embedded in aerial vehicles for their low weight and consumption at an affordable cost.

Specifically, monocular vision presents significant advantages respect other camera configurations (mainly stereo-vision). A single camera does not present the problem of a stereo rig with a fixed baseline between cameras limitating the operational range. But as a drawback the use of a single camera means to face some technical challenges: depth information has to be retrieved with many frames and, therefore, robust techniques for recovering the feature depth are needed. Some examples of recent works about general monocular SLAM systems that have shown great results are: [19–21].

**Related work:** There are different approaches for implementing monocular SLAM systems applied to aerial vehicles which some of them are variations of more general methods. In [22] SURF visual features are used within an EKF-based (Extended Kalman Filter) SLAM scheme. In this case, features are initialized into the state by using the undelayed inverse depth (UID) method, proposed in [23]. In [24] an homography-based SLAM approach is proposed. In this case homography-based techniques are used to compute the UAV relative translation and rotation by means of the images. The visual odometer is then integrated into the SLAM scheme via an EKF. The work in [25] also uses an homography-based method for estimating the motion of the vehicle. The computed motion is used as input of an EKF-SLAM that fuses inertial measurements. Initialization of features is done by the UID method. In [26], an EKF-based approach is proposed where feature depth is computed by triangulation between visual correspondences using SIFT descriptors. In [27] a method that estimates depth and vehicle states, by exploiting the orthogonality of indoor environments, is proposed. The SLAM formulation used in that work is the FastSLAM algorithm proposed in [28]. In [29] a fully navigation scheme (control and estimation) is proposed. In this case the Parallel Tracking and Mapping (PTAM) algorithm, described in [30], is used for implementing the SLAM system. In [31] an EKF scheme is embedded into the PTAM algorithm for fusing IMU (inertial measurement unit) data, in order to recover the absolute scale of estimations. In [32] a variation of the PTAM algorithm is proposed to be applied in environments with very few visual features. In [33] another variation of the PTAM algorithm is proposed. A Bayesian filter that explicitly models outlier measurements is used to estimate the depth of feature locations: a 3D point is only inserted in the map when the corresponding depth-filter has converged.

As it can appreciated from the above approaches in literature, most of them are filter-based methods, Keyframe methods (PTAM), or a mixture of them. While Keyframe methods are shown to give accurate results when the availability of computational power is enough, filtering-based SLAM methods might be beneficial if limited processing power is available [15].

**Objectives and contributions:** In this work authors propose a new filter-based monocular SLAM scheme. The method presented in this research has been designed for taking advantage of hardware resources commonly available in this kind of platforms. The performance of the method is validated by means of experiments with real data carried out in unstructured outdoor environments. An extensive comparative study is presented for contrasting the operative and effectiveness of this proposal respect to other relevant methods. One of the contributions of this work is to present a novel technique for estimating the features depth. The proposed approach is based on a stochastic technique of triangulation. While this technique is inspired in a previous authors’ work [34], crucial and contributive modifications have been introduced in order to accommodate it to the particularities of the current application:

- In this work, the camera is mounted over a servo-controlled gimbal that counteracts the changes in attitude of the quadcopter. Due to the above assumption, the overall problem is simplified and it is focused on the position estimation of the MAV. Also, the tracking process of visual features is made easier due to the stabilized video.
- Instead of using an external pattern of known dimensions, in this work the GPS signal is used during a short initial period of time for recovering the metric scale of the estimates.
- Features are directly parametrized in their euclidean form, instead of the inverse depth parametrization. The consequence is a reduction of the computational cost of the filter due to the reduction of the dimension of the system state.
- A novel technique for the tracking process of candidate points is proposed. In this case the search of visual features is limited to regions of the image circumscribed by ellipses derived from epipolar constraints. The consequence is an improvement in the execution time.

Compared with other methods presented in literature, one of the contributions of this work is to demonstrate that the integration of very noisy GPS measurements into the system for an initial short period is enough to initialize the metric scale. For example in [35] the monocular scale factor is retrieved from a feature pattern with known dimensions. In [29] and [36], the map is initially set by hand, by aligning the first estimates with the ground-truth in order to get the scale of the environment. Additionally, the proposed approach is simpler when compared with similar approaches, because the estimation of the camera orientation is avoided by using the servo-controlled gimbal. In [26] feature depth is computed by direct triangulation between visual correspondences using SIFT descriptors. In this work, a novel technique, which is based on patch-correlation, is used for the tracking process of candidate points. It is well known that local descriptors like SIFT or SURF are more robust that the use of patch-correlation techniques for matching visual features. Nevertheless, the stabilized video and the stochastic nature of the whole initialization method makes reliable the technique proposed in this work for tracking candidate points, with the implicit gain in terms of computational cost.

Perhaps, the most extended technique that is used for initializing map features in EKF-SLAM is the UID based methods (e.g. [22, 25]). Nevertheless, the comparison study presented in this work shows that the proposed method can surpass the UID method in terms of accuracy and computational time, at least for the kind of application studied.

**Paper outline:** Section 2 states the problem description and assumptions. Section 3 describes the proposed method in a detailed manner. In Section 4 experimental results are shown together with a comparative study and the discussion about those results and, finally, Section 5 presents the conclusions of this work.

## 2 Assumptions

**Fig 1:**
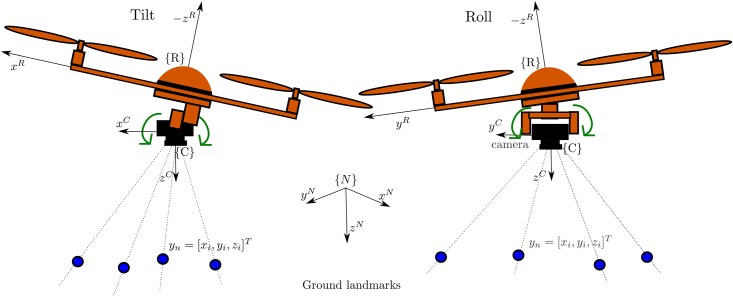
Coordinate systems: the local tangent frame is used as the navigation reference frame ^*N*^. Monocular camera is mounted over a servo-controlled gimbal that counteracts the changes in attitude of the quadcopter.

The platform considered in this work is a quadrotor with free movements in any direction in $R^{3}\times SO\left(3\right)$, shown in Fig 1. However, it is important to highlight that the proposed monocular SLAM method could be applied to other kind of platforms. The proposed method is mainly intended for local autonomous vehicle navigation. In this case, the local tangent frame is used as the navigation reference frame. Thus, the initial position of the vehicle defines the origin of the navigation coordinates frame. The navigation system follows the NED (North, East, Down) convention. The magnitudes expressed in the navigation and in the camera frame are denoted respectively by the superscripts ^*N*^ and ^*C*^. All the coordinate systems are right-handed defined. It is also assumed that the location of the origin of camera frame respect to other elements of the quadcopter (e.g. GPS antenna) is known and fixed. In this case, the position of the origin of the vehicle can be computed from the estimated location of the camera.

In aerial vehicles applications, the attitude estimation is well handled by available systems (e.g. [37] and [38]), therefore, this work will focus in position estimation. Also, it is assumed that the monocular camera is mounted over a servo-controlled gimbal (see Fig 1). This kind of accessory, used mainly for stabilizing video capture, has become very common in aerial applications. In our case, the gimbal is configured in order to counteract the changes in attitude of the quadcopter, and therefore stabilizing the orientation of the camera towards the ground (down axis in NED coordinates). The above consideration has two important consequences: i) the tracking process of visual features is made easier due to the stabilized video, ii) the orthogonal matrix *R*^*CN*^, defining the rotation of the camera frame to the navigation frame, is assumed to be known.

An standard monocular camera is considered. In this case, a central-projection camera model is assumed. The image plane is located in front of the camera’s origin where a non-inverted image is formed. The camera frame ^*C*^ is right-handed with the *z*-axis pointing to the field of view.

The $R^{3}\Rightarrow R^{2}$ projection of a 3D point located at *p*^*N*^ = (*x*, *y*, *z*)^*T*^ to the image plane *p* = (*u*, *v*) is defined by:

$$u=\frac{x^{\prime }}{z^{\prime }}\;\;\;\;\;v=\frac{y^{\prime }}{z^{\prime }}$$ (1)

Let *u* and *v* be the coordinates of the image point *p* expressed in pixel units, and:

$$\left[\begin{array}{c} x^{\prime }\\ y^{\prime }\\ z^{\prime } \end{array}\right]=\left[\begin{array}{ccc} f & 0 & u_{0}\\ 0 & f & v_{0}\\ 0 & 0 & 1 \end{array}\right]p^{C}$$ (2)

Let *p*^*C*^ be the same 3D point *p*^*N*^, but expressed in the camera frame ^*C*^ by *p*^*C*^ = *R*^*NC*^ *p*^*N*^. Let *R*^*NC*^ be the rotation matrix that allows to transform from the navigation frame ^*N*^ to the camera frame ^*C*^. Also, it is fulfilled that *R*^*NC*^ = (*R*^*CN*^)^*T*^, and *R*^*CN*^ is known by the use of the gimbal.

Inversely, a directional vector $h^{C}={\left[h_{x}^{C},h_{y}^{C},h_{z}^{C}\right]}^{T}$ can be computed from the image point coordinates *u* and *v* as

$$h^{C}\left(u,v\right)={\left[\frac{u_{0}-u}{f},\frac{v_{0}-v}{f},1\right]}^{T}$$ (3)

Vector *h*^*C*^ points from the camera optical center position to the 3D point location and it can be expressed in the navigation frame by *h*^*N*^ = *R*^*CN*^ *h*^*C*^. Note that for the $R^{2}\Rightarrow R^{3}$ mapping case, defined in Eq 3, depth information is lost.

The distortion caused by the camera lens is considered through the model described in [39]. Using the former model (and its inverse form), undistorted pixel coordinates (*u*, *v*) can be obtained from (*u*_*d*_, *v*_*d*_) and conversely. In this case, it is assumed that the intrinsic parameters of the camera are already known: focal length *f*, principal point (*u*_0_, *v*_0_), and radial lens distortion *k*_1_, …, *k*_*n*_.

## 3 Method description

### 3.1 Problem description

The main goal of the proposed method is to estimate the following system state *x*:

$$x={\left[x_{v},y_{1},y_{2},...,y_{n}\right]}^{T}$$ (4)

where *x*_*v*_ represents the state of the camera-quadcopter, and *y*_*i*_ represents the location of the *i*-th feature point in the environment. At the same time, *x*_*v*_ is composed of:

$$x_{v}={\left[r^{N},v^{N}\right]}^{T}$$ (5)

Let *r*^*N*^ = [*p*_*x*_, *p*_*y*_, *p*_*z*_] represent the position of the vehicle (camera) expressed in the navigation frame. Let *v*^*N*^ = [*v*_*x*_, *v*_*y*_, *v*_*z*_] denote the linear velocity of the robot expressed in the navigation frame. The location of a feature *y*_*i*_ is parametrized in its euclidean form:

$$y_{i}={\left[p_{x_{i}}p_{y_{i}},p_{z_{i}}\right]}^{T}$$ (6)

### 3.2 Prediction

The work presented in this paper is motivated by the application of monocular SLAM to small aerial vehicles. In this case, and due to limited resources commonly available in this kind of applications, the filtering-based SLAM methods seem to be still more appropriate than Keyframe methods. Moreover, filtering-based methods are better suited for incorporating, in a simple manner, additional sensors to the system. In this sense, most robotic applications make use of multiple sensor inputs.

The architecture of the system is defined by the typical loop of prediction-updates steps in the EKF in direct configuration, where the EKF propagates the vehicle state as well as the feature estimates. In this case, the camera-vehicle system state *x*_*v*_ takes a step forward by the following simple model:

$$\left\{\begin{array}{c} r_{k+1}^{N}=r_{k}^{N}+v_{k}^{N}\Delta t\\ v_{k+1}^{N}=v_{k}^{N}+V^{N} \end{array}\right.$$ (7)

At every step, it is assumed that there is an unknown linear velocity with acceleration zero-mean and known-covariance Gaussian processes *σ*_*a*_, producing an impulse of linear velocity: $V^{N}=\sigma_{a}^{2}\Delta t$.

It is assumed that the map features *y*_*i*_ remain static (rigid scene assumption) so *x*_*k*+1_ = [*x*_*v*(*k*+1)_, *y*_1(*k*)_, *y*_2(*k*)_, …, *y*_*n*(*k*)_]^*T*^.

The state covariance matrix ***P*** takes a step forward by:

$$P_{k+1}=\nabla F_{x}P_{k}\nabla F_{x}^{T}+\nabla F_{u}Q\nabla F_{u}^{T}$$ (8)

where Q and the Jacobians ∇*F*_*x*_, ∇*F*_*u*_ are defined as:

$$\nabla F_{x}=\left[\begin{array}{cc} \frac{\partial f_{v}}{\partial x_{v}} & 0_{6\times n}\\ 0_{n\times 6} & I_{n\times n} \end{array}\right],\nabla F_{u}=\left[\begin{array}{cc} \frac{\partial f_{v}}{\partial u} & 0_{6\times n}\\ 0_{n\times 3} & 0_{n\times n} \end{array}\right],Q=\left[\begin{array}{cc} U & 0_{3\times n}\\ 0_{n\times 3} & 0_{n\times n} \end{array}\right],$$ (9)

Let $\frac{\partial f_{v}}{\partial x_{v}}$ be the derivatives of the equations of the nonlinear prediction model (Eq 7) with respect to the robot state *x*_*v*_. Let $\frac{\partial f_{v}}{\partial u}$ be the derivatives of the nonlinear prediction model with respect to the system input *u*. Uncertainties are incorporated into the system by means of the process noise covariance matrix $U=\sigma_{a}^{2}I_{3\times 3}$, through parameter $\sigma_{a}^{2}$.

### 3.3 Detection of candidate points

**Fig 2:**
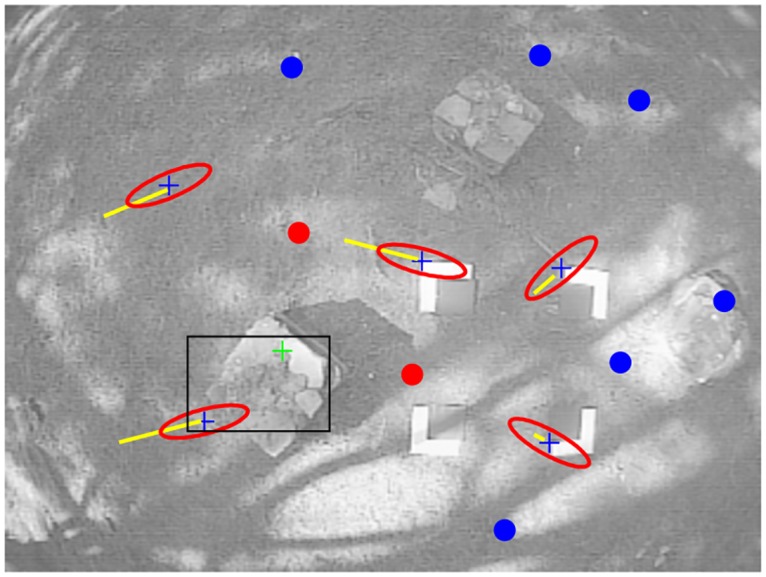
New candidate points are randomly detected in image regions that are empty of map features or candidate points being tracked. In this frame, the black rectangle indicates the current search region where new candidate points have been detected (green cross mark). In order to speed up the tracking process of candidate points, a search region is established constrained by ellipses (in red) aligned with the epipolar lines (in yellow). Candidate points being tracked are indicated by blue cross marks. Visual features already mapped are indicated by dots. Red dots indicate unsuccessfully matches.

The proposed method states that a minimum number of features *y*_*i*_ is considered to be predicted appearing in the image, otherwise new features should be added to the map. In the latter case, new points are detected in the image through a random search. For this purpose, Shi-Tomasi corner detector [40] is applied, but other detectors could also be used. These points in the image, which are not added yet to the map, are called candidate points (see Fig 2). Only image areas free of both, candidate points and mapped features, are considered for detecting new points with the saliency operator.

At the *k*-th frame, when a visual feature is detected for the first time, the following entry *c*_*l*_ is stored in a table:

$$c_{l}=\left[{\left(t_{c_{0}}^{N}\right)}^{T},\theta_{0},\phi_{0},P_{y_{i}},u,v\right]$$ (10)

where $y_{c_{i}}=\left[{\left(t_{c_{0}}^{N}\right)}^{T},\theta_{0},\phi_{0}\right]$ models a 3D semi-line, defined on one side by the vertex ${\left(t_{c_{0}}^{N}\right)}^{T}$, corresponding to the current optical center coordinates of the camera expressed in the navigation frame, and pointing to infinite on the other side with azimuth and elevation *θ*_0_ and *ϕ*_0_, respectively, being:

$$\begin{array}{c} \theta_{0}=\text{atan}2\left(h_{y}^{N},h_{x}^{N}\right)\\ \phi_{0}=\text{acos}\left(\frac{h_{z}^{N}}{\sqrt{{\left(h_{x}^{N}\right)}^{2}+{\left(h_{y}^{N}\right)}^{2}+{\left(h_{z}^{N}\right)}^{2}}}\right) \end{array}$$ (11)

where $h^{N}={\left[h_{x}^{N},h_{y}^{N},h_{z}^{N}\right]}^{T}$ is computed as indicated in Section 2. Let *P*_*y*_*i*__ be a 5 × 5 covariance matrix which models the uncertainty of *y*_*i*_. Therefore *P*_*y*_*i*__ = *JPJ*^*T*^, where *P* is the system covariance matrix and *J* is the Jacobian matrix formed by the partial derivatives of the function *y*_*c*_*i*__ = *h*(*x*, *z*_*uv*_) with respect to [*x*, *z*_*uv*_]^*T*^. Let [*u*, *v*] be the location in the image of the candidate point.

Also, a *p* × *p*-pixel window, centered in [*u*, *v*] is extracted and linked to the corresponding candidate point.

### 3.4 Tracking of candidate points

**Fig 3:**
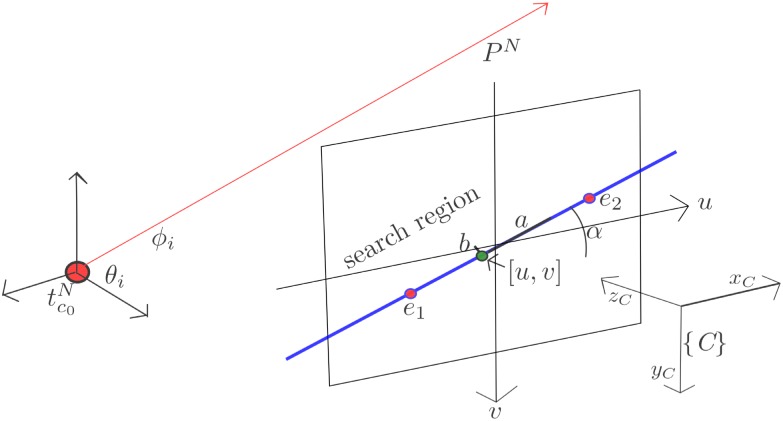
The established search region for matching candidate points is constrained by ellipses aligned with the epipolar line.

At every subsequent frame *k* + 1, *k* + 2…*k* + *n*, the location of candidate points is tracked. In this case, a candidate point is predicted to appear inside an elliptical region *S* centered in the point [*u*, *v*], taken from *c*_*l*_, see Fig 3.

In order to optimize the speed of the search, the major axis of the ellipse is aligned with the epipolar line defined by image points *e*_1_ and *e*_2_.

The epipole *e*_1_ is computed by projecting $t_{c_{0}}^{N}$, which is stored in *c*_*l*_, to the current image plane by Eqs 1 and 2. The point *e*_2_ is computed by projecting the 3D point *p*^*N*^ defined by the data stored in *c*_*l*_, through Eqs 1 and 2 also, but assuming a depth equal to one (*d* = 1). In this case, *p*^*N*^ models a 3D point located at:

$$p^{N}=t_{c}^{N}+d\left(m\left(\theta_{i},\phi_{i}\right)\right)$$ (12)

where *m*(*θ*_*i*_, *ϕ*_*i*_) is a directional unitary vector defined by:

$$m\left(\theta_{i},\phi_{i}\right)={\left(\cos\theta_{i}\sin\phi_{i},\sin\theta_{i}\sin\phi_{i},\cos\phi_{i}\right)}^{T}$$ (13)

The orientation of the ellipse *S*_*c*_ is determined by *α*_*c*_ = atan2(*e*_*y*_, *e*_*x*_) where *e* = *e*_2_ − *e*_1_ and *e*_*y*_, *e*_*x*_ represent the *y* and *x* coordinates respectively of *e*. The size of the ellipse *S*_*c*_ is determined by its major and minor axis, respectively *a* and *b*. In this case *a* and *b* are free parameters constrained to *b* ≪ *a*.

The ellipse *S*_*c*_ is represented in its matrix form by:

$$\begin{array}{c} S_{c}=R_{c}\left[\begin{array}{cc} a & 0\\ 0 & b \end{array}\right]R_{c}^{T}\\ R_{c}=\left[\begin{array}{cc} \cos\alpha_{c} & -\sin\alpha_{c}\\ \sin\alpha_{c} & \cos\alpha_{c} \end{array}\right] \end{array}$$ (14)

The ellipse *S*_*c*_ represents a probability region where the candidate point must lie in the current frame. In this case, patch cross-correlation is applied over all the image locations [*u*_*S*_, *v*_*S*_] within the search region. If the score of a location [*u*_*S*_, *v*_*S*_], determined by the best cross-correlation between the candidate patch and the *n* patches defined by the region of search, is higher than a threshold, then this pixel location [*u*_*S*_, *v*_*S*_] is considered as the current candidate point location. Thus, *c*_*l*_ is updated with [*u*, *v*] = [*u*_*S*_, *v*_*S*_].

At this stage, there is not yet reliable information about the depth of candidate points. For this reason, it is difficult to determine an optimal size of the search ellipse. In this case, the parameter *a* is chosen empirically in function of the particularities of the application as the velocity of the vehicle and the frame rate of the video. In this work, good results were found with a value of *a* = 20 pixels.

**Fig 4:**
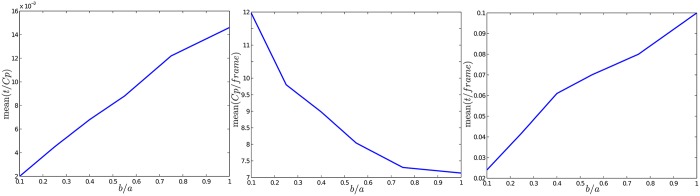
Variation of the relation between ellipse *S*_*c*_ axes (*b*/*a*). Left plot: average tracking time for a candidate point. Middle plot: average number of candidate points being tracked at each frame. Right plot: average total time per frame.

Nevertheless, the effects obtained by the variation of the relation of (*b*/*a*), which determines the proportion of the ellipse, can be investigated. In this case, some experimental results were obtained using the same methodology described in Section 4. The results can be summarized as follows (see Fig 4):

- As the ellipse tends to be a circle, the time required to track a candidate point increases considerably (left plot).
- When the ellipse tends to be a circle the number of candidate points being tracked is lower (middle plot). This is because when the ellipse is too thin, some candidate points are lost and new ones must be detected.
- When the parameter *b* is chosen in order to define a very thin ellipse, the total time required for the whole tracking process of candidate points is much lower (right plot).

Based on the above results, the value of parameter *b* is recommended to be ten times lower than *a*.

It is important to note that no extra effort is put in order to obtain a more robust descriptor. There are two main reasons for supporting this approach: i) The method proposed for tracking the candidate points is applied only during an initial short period when a new visual feature is detected. During this initial period, prior to the initialization of the candidate point as a new map feature, some information about the feature depth is gathered. ii) Different from the general problem of the monocular SLAM, the stabilized video also makes easier the tracking process of candidate points.

### 3.5 Feature initialization

**Fig 5:**
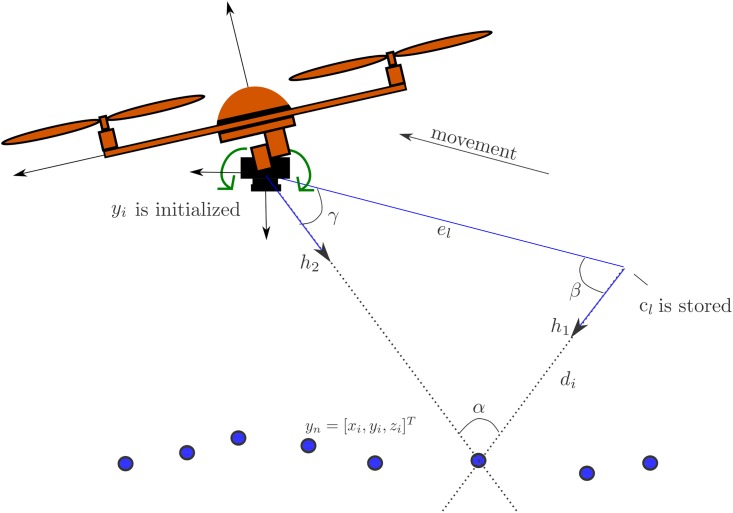
An hypothesis of depth *d*_*i*_ of a candidate point is computed by triangulation between the first location when the point was detected and the current location of the aerial vehicle.

Depth information cannot be obtained in a single measurement when bearing sensors (e.g. a projective camera) are used. To infer the depth of a feature, the sensor must observe this feature repeatedly as this sensor moves freely through its environment, estimating the angle from the feature to the sensor center. The difference between those angle measurements is the parallax angle. Actually, parallax is the key that allows estimating features depth. In case of indoor sequences, a displacement of centimeters could be enough to produce parallax; on the other hand, the more distant the feature, the more the sensor has to travel to produce parallax (see Fig 5).

Every time that a new image location *z*_*uv*_ = [*u*, *v*] is obtained for a candidate point *c*_*l*_, an hypothesis of depth *d*_*i*_ is computed by:

$$d_{i}=\frac{\|e_{l}\|\sin\gamma }{\sin\alpha }$$ (15)

Let *α*_*i*_ = *π* − (*β* + *γ*) be the parallax. Let $e_{l}=t_{c_{0}}^{N}-t_{c}^{N}$ indicate the displacement of the camera from its first observation to its current position with:

$$\beta =\cos^{-1}\left(\frac{h_{1}\cdot e_{l}}{\|h_{1}\left\|\right\|e_{l}\|}\right)\;\;\;\;\;\gamma =\cos^{-1}\left(\frac{h_{2}\cdot -e_{l}}{\|h_{2}\left\|\right\|e_{l}\|}\right)$$ (16)

Let *β* be the angle defined by *h*_1_ and *e*_*l*_. Let *h*_1_ be the normalized directional vector *m*(*θ*, *ϕ*) = (cos *θ* sin *ϕ*, sin *θ* sin *ϕ*, cos *ϕ*)^*T*^ computed taking *θ*_0_, *ϕ*_0_ from *c*_*l*_, and where *γ* is the angle defined by *h*_2_ and −*e*_*l*_. Let *h*_2_ = *h*^*N*^ be the directional vector pointing from the current camera optical center to the feature location computed as indicated in Section 2 from the current measurement *z*_*uv*_.

At each step, the hypothesis of depth *d*_*i*_ is low-pass filtered because the depth estimated by triangulation varies considerably, specially for low parallax. In previous authors’ work [34] is demonstrated that only a few degrees of parallax is enough to reduce the uncertainty in depth estimations.

When parallax *α*_*i*_ is greater than a specific threshold (*α*_*i*_ > *α*_*min*_) a new feature *y*_*new*_ = [*p*_*x*_*i*__, *p*_*y*_*i*__, *p*_*z*_*i*__]^*T*^ is added to the system state vector *x*:

$$x_{new}={\left[x_{old};y_{new}\right]}^{T}$$ (17)

where

$$y_{new}=t_{c_{0}}^{N}+m\left(\theta_{0},\phi_{0}\right)d_{i}$$ (18)

The system state covariance matrix *P* is updated by:

$$P_{new}=\left[\begin{array}{cc} P_{old} & 0\\ 0 & P_{y_{new}} \end{array}\right]$$ (19)

Let *P*_*y*_*new*__ be the 3 × 3 covariance matrix which models the uncertainty of the new feature *y*_*new*_, and:

$$P_{y_{new}}=J\left[\begin{array}{cc} P_{y_{i}} & 0\\ 0 & \sigma_{d}^{2} \end{array}\right]J^{T}$$ (20)

In Eq 20, *P*_*y*_*i*__ is taken from *c*_*l*_. Let $\sigma_{d}^{2}$ be a parameter modelling the uncertainty of process of depth estimation. Let *J* be the Jacobian matrix formed by the partial derivatives of the function *y*_*new*_ = *h*(*c*_*l*_, *d*_*i*_) with respect to ${\left[{\left(t_{c_{0}}^{N}\right)}^{T},\theta_{0},\phi_{0},d_{i}\right]}^{T}$.

### 3.6 Visual updates and map management

The process of tracking visual features *y*_*i*_ is conducted by means of an active search technique [41]. In this case, and in different way from the tracking method described in subsection 3.4, the search region is defined by the innovation covariance matrix *S*_*i*_, where $S_{i}=\nabla H_{i}P_{k+1}\nabla H_{i}^{T}+R_{i}$.

Assuming that for the current frame, *n* visual measurements are available for features *y*_1_, *y*_2_, …, *y*_*n*_, then the filter is updated with the Kalman update equations as:

$$\left\{\begin{array}{c} x_{k}=x_{k+1}+K\left(z-h\right)\\ P_{k}=P_{k+1}-KSK^{T}\\ K=P_{k+1}\nabla H^{T}S^{-1}\\ S=\nabla HP_{k+1}\nabla H^{T}+R \end{array}\right.$$ (21)

where *z* = [*z*_*uv*_1__, *z*_*uv*_2__, …, *z*_*uv*_*n*__]^*T*^ is the current measurement vector. Let *h* = [*h*_1_, *h*_2_, …, *h*_*n*_]^*T*^ be the current prediction measurement vector. The measurement prediction model *h*_*i*_ = (*u*, *v*) = *h*(*x*_*v*_, *y*_*i*_) has been defined in Section 2. Let *K* be the Kalman gain. Let *S* be the innovation covariance matrix. Let ∇*H* = [∇*H*_1_, ∇*H*_2_, …, ∇*H*_*n*_]^*T*^ be the Jacobian formed by the partial derivatives of the measurement prediction model *h*(*x*) with respect to the state *x*, as:

$$\nabla H_{i}=\left[\frac{\partial h_{i}}{\partial x_{v}},...0_{2\times 3}...,\frac{\partial h_{i}}{\partial y_{i}},...0_{2\times 3}...\right]$$ (22)

Let $\frac{\partial h_{i}}{\partial x_{v}}$ be the partial derivatives of the equations of the measurement prediction model *h*_*i*_ with respect to the robot state *x*_*v*_. Let $\frac{\partial h_{i}}{\partial y_{i}}$ be the partial derivatives of *h*_*i*_ with respect to feature *y*_*i*_. Note that $\frac{\partial h_{i}}{\partial y_{i}}$ has only a nonzero value at the location (indexes) of the observed feature *y*_*i*_. Let $R=\left(I_{2n\times 2n}\right)\sigma_{uv}^{2}$ be the measurement noise covariance matrix.

A SLAM framework that works reliably in a local way can easily be applied to large-scale problems using different methods, such as sub-mapping, graph-based global optimization [15] or global mapping [42]. Therefore, in this work, large-scale SLAM and loop-closing are not considered. Nevertheless these problems have been intensively studied in the past. Candidate points whose tracking process fails are pruned from the system. In a similar way, visual features with high percentage of mismatching are removed from the system state and covariance matrix.

### 3.7 Metric scale and System initialization

**Fig 6:**
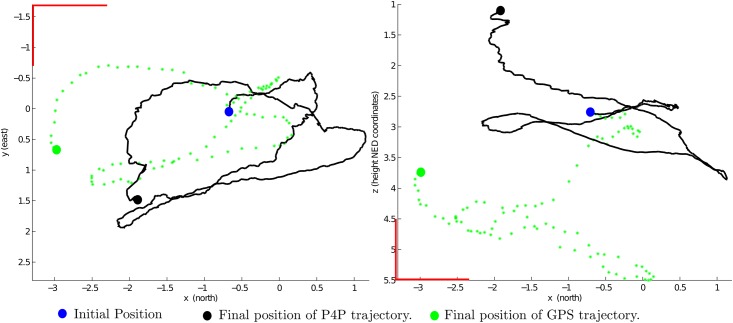
Example of the GPS position measurements obtained for a flight trajectory. Top view (left plot) and lateral view (right plot). In this case, the flight trajectory has been computed using the P4P method described in the Appendix. Observe the error drift in GPS readings.

Even when GPS signal is available, the problem of position estimation could not be solved for some specific scenarios, for instance in an application requiring performing precision manoeuvres in a complex environment. In this case, and due to several sources of error, the position obtained with a GPS can vary even for meters in a matter of seconds for a static location [43]. In such a scenario, the use of GPS readings, smoothed or not, as feedback signal of the control system can be unreliable because the control is not able to discriminate between sensor noise or actual small movements of the vehicle (see Fig 6).

On the other hand, in a robotics context, obtaining the metric scale of the world can be a tough requirement. However, one of the most challenging aspects of working with monocular sensors has to do with the impossibility of directly recovering the metric scale of the world. If no additional information is used, and a single camera is used as the solely source of data to the system, the map and trajectory can only be recovered without metric information [14]. In this case, neither monocular vision nor GPS are suitable to be used separately for navigation purposes.

In this work, noisy data obtained from the GPS is incorporated into the system at the beginning in order to incorporate the metric information of the environment. After some initial period of convergence, where the system is considered to be in a initialization mode, the system can operate relying only on visual information.

Position measurements obtained from the GPS are modelled by:

$$y_{r}=r^{N}+v_{r}$$ (23)

where *v*_*r*_ is a Gaussian white noise with PSD $\sigma_{r}^{2}$; and *r*^*N*^ has been already defined in Eq 7.

Commonly, position measurements are obtained from GPS devices in geodetic coordinates (*latitude*, *longitude* and *height*). Therefore, in Eq 23 it is assumed that GPS position measurements have been previously transformed to their corresponding local tangent frame coordinates. It is also assumed that the offset between the GPS antenna and the vehicle frame has been taken into account in the previous transformation.

For system updates, the simple measurement model *h*_*r*_ = *h*(*x*_*v*_) is used:

$$h_{r}={\left[p_{x},p_{y},p_{z}\right]}^{T}$$ (24)

In the next Section, the demonstration that the proposed method is robust enough to be initialized with noisy GPS measurements will be shown.

## 4 Experimental Results

### 4.1 Experimental setup

**Fig 7:**
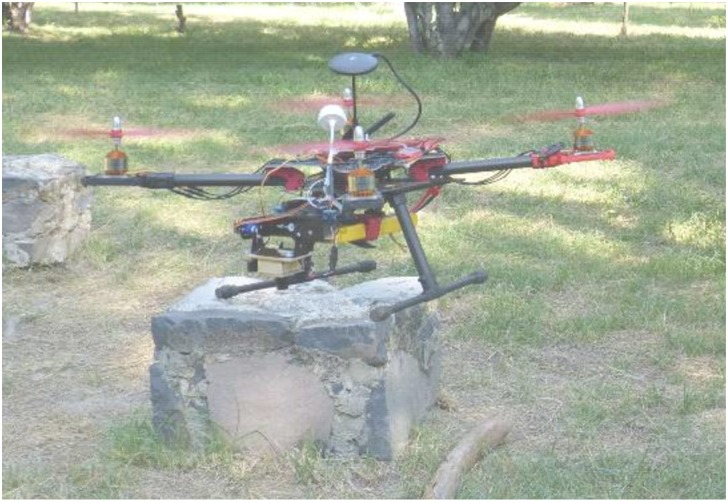
Data obtained from the sensors of a radio-controlled quadrotor has been used for testing the proposed method. A urban park was used as flight field.

In Fig 7 is shown the vehicle that authors used to obtain real data for experiments, the platform is a customized quadrotor. Such a platform uses an Ardupilot unit, [44], as flight controller. As main sensors, the platform is equipped with a radio telemetry unit (3DR at 915MHz), GPS unit (NEO-M8N), camera (DX201 DPS) with wide angle lens and a video transmitter (at 5–8 GHz). The camera is mounted over a very low-cost gimbal which is servo-controlled by standard servos. During the experiments, the quadrotor has been controlled by radio in a manual way.

For capturing sensor data and digitalized video from the vehicle a software application has been built by authors in C++ language. The protocol used for reception/transmission is MAVLINK protocol [45]. GPS and AHRS (Attitude and Heading Reference System) data are synchronized between them and recorded in a database for their study. Video frames have been acquired at a resolution of 320x240 gray scale pixels at 25 *fps*. All the experiments have been performed in an outdoor park with trees, which surface is almost flat with grass and some dirt areas. Flight observations include some plants and small structured parts. In average 9–10 GPS satellites are visible at the same time. Finally, a MATLAB implementation of the proposed method was executed offline over the dataset in order to estimate the flight trajectory and the map of the environment. In experiments, for evaluating the performance of the proposed method, the technique P4P described in the Appendix was used in order to have an external reference of the flight trajectory. In the following website reader can download the different files containing all the data collected by robot sensors. This data has been used by authors to perform the experiments contained in this research paper (https://figshare.com/articles/Experiments/4029111).

### 4.2 Flight trajectories

**Fig 8:**
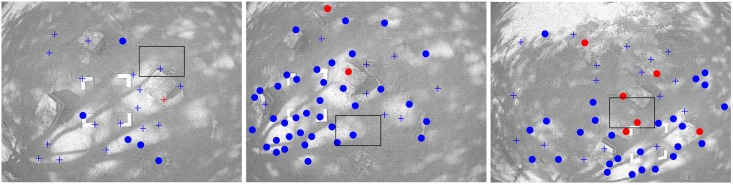
Frames taken from flight *F*_*a*_: instant *t* = 2.84*s* (left plot), instant *t* = 10.23*s* (middle plot) and instant *t* = 30.11*s* (right plot). Candidate points being tracked are indicated by blue-cross marks. Visual features already mapped are indicated by dots. Red dots indicate unsuccessfully matches. Also note the four marks used for computing the external P4P flight trajectory.

**Fig 9:**
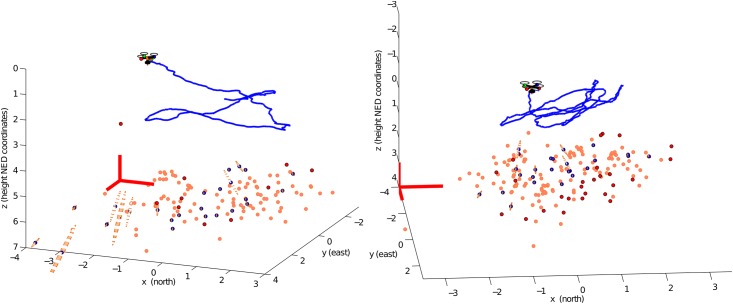
Estimated map and trajectory 3D plots obtained with the proposed delayed monoSLAM method: flight *F*_*a*_ (left plot) and flight *F*_*b*_ (right plot). Uncertainty in features position is indicated by 3D ellipses. Physical structure of the environment is partially recovered observing visual features.

Two different flight trajectories (*F*_*a*_ and *F*_*b*_) were performed over the test field. In both cases, an initial period of 5 seconds (from *t* = 0*s* to *t* = 5*s*) of flight was considered for initialization purposes as it was explained in section 3.7. Fig 8 shows some frames of the video recorded in flight *F*_*a*_. At the beginning of the trajectory (left plot), at instant *t* = 2.84*s*, the first features are added to the system state. Note that at this moment, most of the tracked points are considered as candidate points. At instant *t* = 10.23*s* (middle plot), the system is operating relying only on visual information for estimating the position of the quadcopter and the map of the environment. The right plot shows a frame at instant *t* = 30.11*s*. Fig 9 shows a 3D perspective of the estimated map and trajectory for both flight trajectories *F*_*a*_ and *F*_*b*_. In the next subsection, a more detailed analysis of the experimental results is presented.

### 4.3 Comparative study

A comparative study has been performed in order to gain more insight about the performance of the proposed delayed monoSLAM (DE) method. For this purpose, the DE method has been tested against the popular undelayed inverse depth method (UID), and its variant, the undelayed inverse depth to euclidean method (UID2E). The implementation of the UID and UID2E methods are based respectively on [23] and [46]. The UID and UID2E methods have been chosen because the undelayed inverse depth method has become almost a standard for implementing filter-based monocular-SLAM systems. In experiments, the 1-point RANSAC method [47] has been used for validating the visual matches of map features. In the particular case of the DE method, no extra validation technique was used for the matching process of candidate points. For the DE method, a value of *α*_*min*_ = 5° has been used. For the UID and UID2E methods, values of *ρ*_*ini*_ = 1 and *σ*_*ρ*_*ini*__ = 1 have been used. In general all the methods are tested under the same conditions. Only the parameter $\sigma_{r}^{2}$, used for modelling the uncertainty in GPS readings during the initialization period has slightly been tuned for each method in order to produce a good initial metric convergence.

The search of new candidate points in each frame is conducted in a random manner for the DE method as well as the search of new features in UID and UID2E methods. For this reason, the results of the methods can vary at each run. In this case, in order to have a better statistical appreciation of the performance of each method, 10 Monte Carlo runs have been executed for computing each result.

Tables 1 and 2 show the results obtained respectively for the flight trajectory *F*_*a*_ and *F*_*b*_. The number of visual features being tracked at each frame can affect the performance of monocular SLAM methods. For this reason, the methods have been tested by setting three different values of minimum distance (MD) between the visual features being tracked. In this case, the bigger the value, the lesser the number of visual features that can be tracked. Also, in experiments, features are removed from the system state if they are predicted to appear in the image but are not tracked in 25 periods.

**Table 1:** Results for flight trajectory *F*_*a*_.

Method	MD(*p*)	NIF	NDF	ETF (*s*)	TTE (*s*)	aMAE (*m*)
DE	15	127 ±7*σ*	76 ±4*σ*	.49 ±.11*σ*	268 ±16*σ*	.19 ±.08*σ*
UID	15	302 ±20*σ*	233 ±32*σ*	.62 ±.14*σ*	336 ±19*σ*	.29 ±.18*σ*
UID2E	15	305 ±18*σ*	246 ±23*σ*	.59 ±.11*σ*	323 ±9*σ*	.50 ±.36*σ*
DE	20	90 ±8*σ*	56 ±11*σ*	.34 ±.07*σ*	187 ±5*σ*	.20 ±.11*σ*
UID	20	218 ±11*σ*	175 ±12*σ*	.43 ±.09*σ*	234 ±17*σ*	.31 ±.23*σ*
UID2E	20	216 ±6*σ*	171 ±5*σ*	.40 ±.07*σ*	217 ±6*σ*	.42 ±.30*σ*
DE	25	64 ±2*σ*	39 ±6*σ*	.26 ±.06*σ*	141 ±7*σ*	.23 ±.13*σ*
UID	25	159 ±9*σ*	124 ±5*σ*	.29 ±.06*σ*	162 ±9*σ*	.32 ±.19*σ*
UID2E	25	162 ±9*σ*	133 ±8*σ*	.30 ±.05*σ*	164 ±12*σ*	.53 ±.36*σ*

**Table 2:** Results for flight trajectory *F*_*b*_.

Method	MD(*p*)	NIF	NDF	ETF (*s*)	TTE (*s*)	aMAE (*m*)
DE	15	278 ±13*σ*	210 ±12*σ*	.58 ±.10*σ*	364 ±20*σ*	.29 ±.17*σ*
UID	15	319 ±11*σ*	244 ±13*σ*	.69 ±.16*σ*	428 ±22*σ*	.36 ±.16*σ*
UID2E	15	328 ±8*σ*	245 ±7*σ*	.65 ±.13*σ*	405 ±15*σ*	.52 ±.32*σ*
DE	20	185 ±11*σ*	140 ±8*σ*	.39 ±.06*σ*	242 ±10*σ*	.32 ±.20*σ*
UID	20	217 ±6*σ*	164 ±3*σ*	.45 ±.10*σ*	281 ±16*σ*	.34 ±.15*σ*
UID2E	20	220 ±4*σ*	167 ±2*σ*	.42 ±.08*σ*	260 ±9*σ*	.54 ±.36*σ*
DE	25	143 ±11*σ*	107 ±10*σ*	.29 ±.05*σ*	180 ±11*σ*	.31 ±.19*σ*
UID	25	162 ±4*σ*	121 ±5*σ*	.34 ±.07*σ*	213 ±9*σ*	.35 ±.17*σ*
UID2E	25	170 ±6*σ*	129 ±9*σ*	.32 ±.08*σ*	201 ±15*σ*	.52 ±.32*σ*

Under the above conditions, Tables show the results obtained after applying the three different methods at the end of their trajectories. Some features have been computed for each method (DE, UID and UID2E) such as: i) number of the features initialized into the system state (NIF), ii) number of features deleted from the system state (NDF), iii) execution time per frame (ETF), iv) total time of execution (TTE) and v) average mean absolute error (aMAE) of the vehicle position. For computing the aMAE, the P4P trajectory has been used as an independent reference of the vehicle position (see the Appendix). However, it is important to note that the trajectory obtained by the P4P technique should not be considered as a perfect reference of groundtruth. Despite this consideration, the results obtained still reflect in a very good fashion the performance of every method.

**Fig 10:**
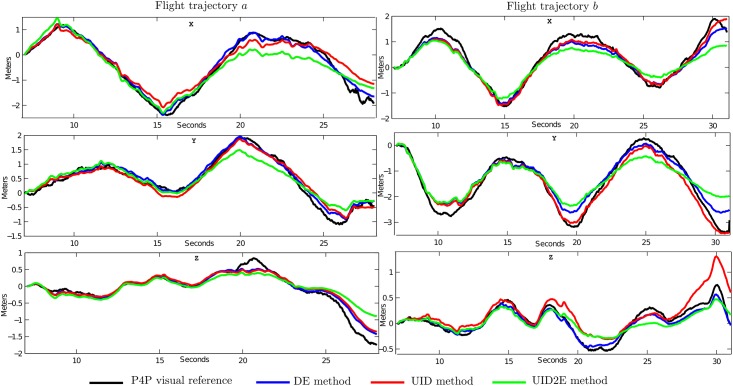
Comparative study of the estimated trajectory of the quadrotor obtained with: i) P4P visual reference (black); ii) DE method (blue); iii) UID method (red); and iv) UID2E method (green). Results are presented in NED coordinates: north (upper plots), east (middle plots) and down (lower plots).

**Fig 11:**
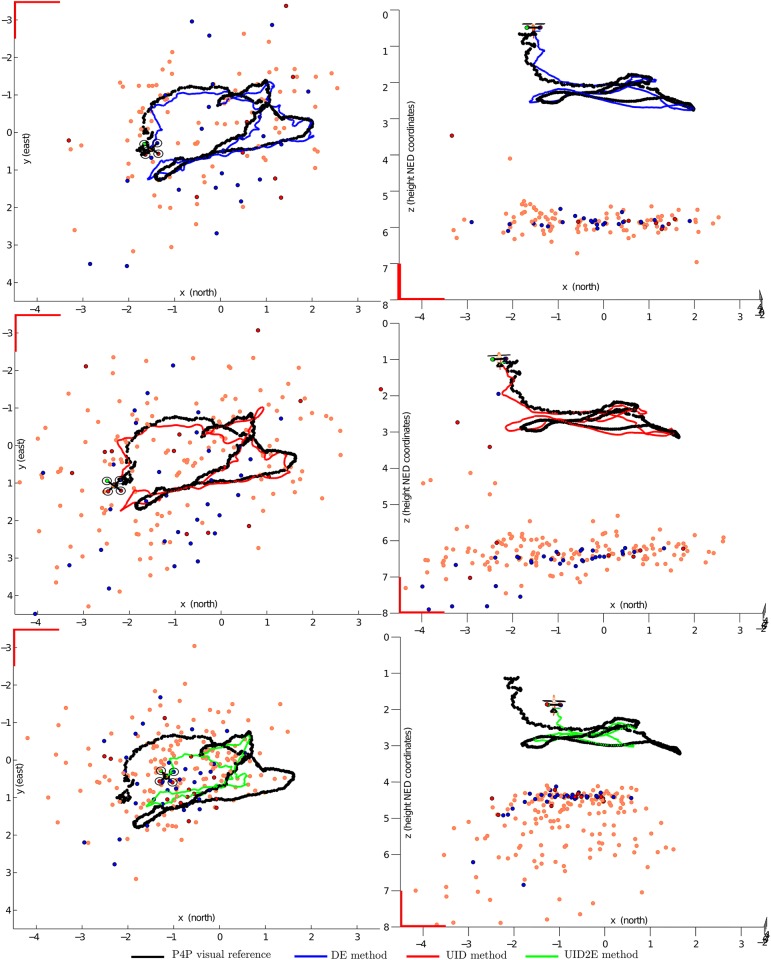
Comparative study of the map and trajectory estimated for the flight *F*_*a*_, with: i) DE method (upper plots); ii) UID method (middle plots); and iii) UID2E method (lower plots). Only top and lateral views are shown (left and right plots respectively). In each case the P4P visual reference is indicated in black. Features deleted from the system state are indicated by small orange spheres. Features contained into the system state, at the end of the trajectory, are indicated by small blue and red spheres. Blue and red spheres mean respectively successful and unsuccessful matches.

Fig 10 shows the estimated position obtained with each method for the flight trajectories *F*_*a*_ and *F*_*b*_. A plot for each NED coordinate (north, east and down) is given. Only the results obtained with a minimum distance between features higher than 20 pixels (MD = 20) are presented. Fig 11 illustrates an example of the estimated map and trajectory that have been obtained with every method. For this figure, top and lateral views are presented.

### 4.4 Discussion

According to the results of the comparative study some implications can be inferred. A slightly variation in the number of features, that are allowed to be tracked at each frame, can significantly affect the number of features that are initialized into the system state. In this case, a reduction of 10 pixels in the MD produces about twice of features initialized. Indeed, an increment of the features initialized into the system state implies an increment of the computational time. On the other hand, theoretically and due to the increment of information available, the increase of tracked features should improve the estimated trajectory. However, results do not show a considerable improvement in this sense. In this case, only with the trajectory *F*_*a*_, a consistent but minor improvement was obtained with the increase of features, but with an increment of about twice the computational time.

Regarding to the average mean absolute error (aMAE) computed for the estimated trajectory of the quadrotor, the DE method has shown consistently slightly better results with respect to the UID method. However, it is important to note that the difference could be within the margin of error of the methodology followed for computing de aMAE. Unfortunately, statistics about this margin of error are not available. For this reason, according to the results DE method can offer at least a similar performance in accuracy with respect to the UID method. On the other hand, the UID2E method shows, in every case, the worst behaviour of all the methods. It is worth noting that, for this application, the UID2E method has exhibited a considerable tendency to drift in the metric scale of the estimations. In Fig 11 (lower plots), it can be clearly appreciated this phenomenon where is specially notorious the degradation in scale of the estimated map.

Regarding to the computational efficiency of the methods, it is clear that the proposed DE method presents the best results. This result can be explained for two reasons: the use of the euclidean parametrization and the use of less but stronger visual features.

In the case of the undelayed methods, the use of the inverse depth (ID) parametrization becomes mandatory due to the nonlinear nature of the measurement equation when features are initialized right after they are detected. On the other hand, ID parametrization requires six parameters instead of three euclidean ones. Therefore, as the number of features increases, with the ID parametrization the length of the state tends to have twice the length that it has with the euclidean parametrization. For the EKF-based approaches, the above ID parametrization has as consequence a well known increment in the computational cost. In this sense, the UID2E method was designed for improving the computational efficiency of the UID method. Features whose depth converge are converted from the ID to euclidean parametrization. Results validate this claim, however, for the application presented in this work, the benefit in computational efficiency is minimal compared with the increase of error drift obtained with the UID2E.

For DE method the period used for candidate points tracking is mainly intended for obtaining information about the features depth, prior to its inclusion into the system state. This fact has also the collateral benefit of pruning weak visual features that fail to be tracked in this period. In contrast to the undelayed methods (UID and UID2E), where all the detected visual features are initialized into the system, delayed methods (DE) initialize less but stronger visual features. This is more evident if the number of initialized features is considered (see Tables 1 and 2), as well as the percentage of deleted features with respect to the number of initialized features: DE = 68%, UID = 77% and UID2E = 78%. These figures mean not only that the undelayed methods initialize a lot of useless visual features, but they also mean that the features initialized with the delayed method are better retained into the system.

## 5 Conclusion

In this work a novel monocular SLAM method with application to a quadcopter has been presented. In this case, a monocular camera is integrated into an UAV in order to provide visual information of the ground. Due to attitude estimation is well handled by available systems for this kind of applications, this research is focused only in position estimation. In order to avoid the need of estimating the camera orientation, a servo-controlled gimbal is used for stabilizing the orientation of the camera towards the ground.

Traditionally, the position estimation of UAVs has been addressed by the use of GPS. However, the GPS is not a fully reliable service as its availability can be limited in urban canyons and is unavailable in indoor environments. Moreover, even when GPS signal is available, the problem of position estimation could not be solved for some specific scenarios, for instance in an application requiring performing precise manoeuvres in a complex environment. Therefore, some additional sensory information should be integrated into the system in order to improve accuracy and robustness. In this context, the use of monocular vision has some advantages in terms of weight, space, energy consumption, or scalability.

On the other hand, two challenging aspects related with monocular sensors have to do with the impossibility of directly recovering the depth of visual features, and the metric scale of the world as well. To address the first aspect, a novel technique for estimating the features depth based in an stochastic technique of triangulation has been presented. Regarding the second aspect, it is assumed that GPS readings are available for some short period at the beginning of the system operation. After this initial period used for incorporating information about the metric scale of the world, the system can operate relying only on visual information for estimating the position of the vehicle.

The performance of the proposed method has been validated by means of experiments with real data carried out in unstructured outdoor environments. To check the contribution of this research, an extensive comparative study is presented for validating the performance of the proposed approach respect similar methodologies. For this kind of aerial application presented in this paper, and according to the experimental results, the proposed method has performed better, in terms of accuracy and execution time, than the UID and UID2E methods.

## 6 Appendix

### 6.1 P4P reference trajectory

Experimental setups in natural outdoor environments can be a challenge for small aerial vehicles. Some difficulty arises with the absence of resources available in laboratories (e.g. Vicon system). In this particular case, for fine flight manoeuvres, the trajectory provided by the GPS is useless to be used as a reference of the actual flight trajectory. In this work, in order to have an independent reference for evaluating the performance of the proposal, the following methodology is proposed.

Four marks are placed in the ground, forming a square of known dimensions, see Fig 8. Each corner is a coplanar point with spatial coordinates [*x*_*i*_, *y*_*i*_, 0] with *i* ∈ 1, …4, and their corresponding four undistorted image coordinates [*u*_*i*_, *v*_*i*_] with *i* ∈ 1…4. Then, for each frame a perspective on 4-point (P4P) technique [48], is applied iteratively in order to compute the relative position of the camera with respect to the known metric reference. At each frame, the image location of the four corners is provided by a simple tracking algorithm designed for this purpose.

The P4P technique used for estimating the camera position, defined by *R*^*CN*^ and *r*^*N*^, is based on [49]. The following linear system is formed with the vector *b* as unknown parameter:

$$\left[\begin{array}{cccccccc} x_{1}f & y_{1}f & 0 & 0 & -u_{1}x_{1} & -u_{1}y_{1} & f & 0\\ 0 & 0 & x_{1}f & y_{1}f & -v_{1}x_{1} & -v_{1}y_{1} & 0 & f\\ : & : & : & : & : & : & : & :\\ x_{4}f & y_{4}f & 0 & 0 & -u_{4}x_{4} & -u_{4}y_{4} & f & 0\\ 0 & 0 & x_{4}f & y_{4}f & -v_{4}x_{4} & -v_{4}y_{4} & 0 & f \end{array}\right]b=\left[\begin{array}{c} u_{1}\\ v_{1}\\ :\\ u_{4}\\ v_{4} \end{array}\right]$$ (25)

where

$$b={\left[\begin{array}{cccccccc} \frac{r_{11}}{r_{3}} & \frac{r_{12}}{r_{3}} & \frac{r_{21}}{r_{3}} & \frac{r_{22}}{r_{3}} & \frac{r_{31}}{r_{3}} & \frac{r_{32}}{r_{3}} & \frac{r_{1}}{r_{3}} & \frac{r_{2}}{r_{3}} \end{array}\right]}^{T}$$ (26)

The linear system represented in Eq 26 is solved for *b* = [*b*_1_ *b*_2_ *b*_3_ *b*_4_ *b*_5_ *b*_6_ *b*_7_ *b*_8_]^*T*^. The camera position is computed from:

$$\begin{array}{cc} R^{CN}=\left[\begin{array}{ccc} r_{3}b_{1} & r_{3}b_{2} & \left(R_{21}R_{32}-R_{31}R_{22}\right)\\ r_{3}b_{3} & r_{3}b_{4} & \left(R_{31}R_{12}-R_{11}R_{32}\right)\\ r_{3}b_{5} & r_{3}b_{6} & \left(R_{11}R_{22}-R_{21}R_{12}\right) \end{array}\right] & r^{N}={\left[\begin{array}{ccc} r_{3}b_{7} & r_{3}b_{8} & r_{3} \end{array}\right]}^{T} \end{array}$$ (27)

where

$$r_{3}=\sqrt{\frac{f^{2}}{b_{1}^{2}+b_{3}^{2}+f^{2}b_{5}^{2}}}$$ (28)

**Fig 12:**
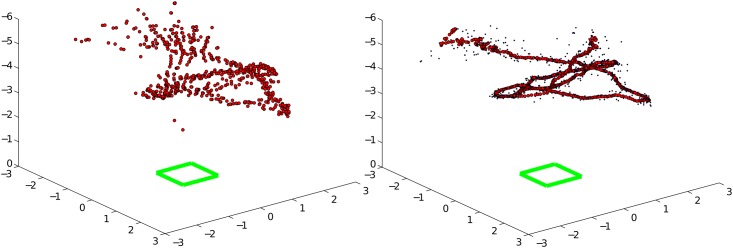
Trajectory obtained by the P4P technique without any filtering (left) and low-pass filtered (right).

In Eq 27 the third column of matrix *R*^*CN*^ is formed by the combinations of the values of first and second column of the same matrix. The results obtained with the above procedure can be very noisy, (see left plot of Fig 12). For this reason, a simple lowpass filter is applied in order to obtain the flight trajectory (right plot, Fig 12).

The P4P trajectory is computed with respect to the metric reference. Trajectories obtained through visual SLAM have their own reference frame. In experiments, both reference frames are aligned in order to make the trajectories coincident at the beginning. In other words, it is assumed that the initial position of the quadcopter is known.
